# Silver Is Not Equal to Silver: Synthesis and Evaluation of Silver Nanoparticles with Low Biological Activity, and Their Incorporation into C_12_Alanine-Based Hydrogel

**DOI:** 10.3390/molecules28031194

**Published:** 2023-01-25

**Authors:** Konrad Kubiński, Kamila Górka, Monika Janeczko, Aleksandra Martyna, Mateusz Kwaśnik, Maciej Masłyk, Emil Zięba, Joanna Kowalczuk, Piotr Kuśtrowski, Mariusz Borkowski, Anna Boguszewska-Czubara, Agnieszka Klimeczek, Oleg M. Demchuk

**Affiliations:** 1 Department of Molecular Biology, Faculty of Medicine, The John Paul II Catholic University of Lublin, Konstantynów 1h, 20-708 Lublin, Poland; 2 Institute of Molecular Physics Polish Academy of Science, M. Smoluchowskiego 17, 60-179 Poznań, Poland; 3 Faculty of Chemistry, Jagiellonian University, Gronostajowa 2, 30-387 Kraków, Poland; 4 Jerzy Haber Institute of Catalysis and Surface Chemistry, Polish Academy of Sciences, Niezapominajek 8, 30-239 Kraków, Poland; 5 Department of Medical Chemistry, Medical University of Lublin, Chodźki 4A, 20-093 Lublin, Poland; 6 Faculty of Physics and Applied Computer Science, AGH University of Science and Technology, Al. Mickiewicza 30, 30-059 Kraków, Poland; 7 Department of Chemistry, Faculty of Medicine, The John Paul II Catholic University of Lublin, Konstantynów 1h, 20-708 Lublin, Poland

**Keywords:** silver nanoparticles, hydrogels, TGA, DLS, XPS, DSC, SEM, Z-potential, drug delivery, biological activity

## Abstract

A new type of silver nanoparticles (AgNPs) was prepared and comprehensively studied. Scanning electron microscopy (SEM) and dynamic light scattering (DLS) analyses indicated that 24 nm AgNPs with narrow size distribution were obtained while Z-potential confirms their good stability. The composites of the obtained AgNPs with nontoxic-nature-inspired hydrogel were formed upon cooling of the aqueous solution AgNPs and C_12_Ala. The thermal gravimetric analysis (TGA) and the differential scanning calorimetry (DSC) do not show significant shifts in the characteristic temperature peaks for pure and silver-enriched gels, which indicates that AgNPs do not strongly interact with C_12_Ala fibers, which was also confirmed by SEM. Both AgNPs alone and in the assembly with the gelator C_12_Ala were almost biologically passive against bacteria, fungus, cancer, and nontumor human cells, as well as zebra-fish embryos. These studies proved that the new inactive AgNPs-doped hydrogels have potential for the application in therapy as drug delivery media.

## 1. Introduction

A huge challenge in modern medicine is the search for alternative and effective solutions to overcome the existing limitations in the diagnosis and treatment of various diseases. This applies mainly to cancer diseases, the treatment of which is associated with the problem of resistance to chemotherapy and with general toxicity of drugs for healthy tissues of the patient [1,2]. Another very important problem are bacterial and fungal infections, which are becoming more and more resistant to existing treatments. A promising idea is to use nanoparticles for this purpose as nanomedicines or delivery systems [3].

Nanotechnology is a field of science that has recently gained great interest in the scientific community due to the wide spectrum of applications in the food, electronics, and cosmetic industries, as well as medicine. The medical use of nanotechnology significantly supports the development of innovative methods of prevention, treatment, diagnosis, and monitoring of diseases [4,5]. In cancer treatment, nanoparticles allow direct delivery of drugs to cancer cells, as well as imaging and early tumor detection and monitoring of treatment effectiveness [6,7]. Nowadays, the potential of application of a large library of nanoparticles, which, thanks to their nanometric dimensions, have unique chemical, physical and biological properties, is being assessed. Nanoparticles of metals such as gold (AuNPs), silver (AgNPs), and copper, as well as silicon dioxide nanoparticles (SiO_2_-NPs), titanium oxide (TiO2-NPs), and zinc oxide (ZnO-NPs), are of great interest [8,9,10,11]. AgNPs have become very popular, which is due to the well-documented antimicrobial properties of silver ions [12]. For example, there are some estimates that the production of silver nanoparticles in the world amounts to about 500 tons per year [13,14]. Silver nanoparticles are widely used, due to their antibacterial and antifungal properties, against a fairly wide spectrum of microbial species [12,15,16,17]. This is possible due to the suspicion that AgNPs may cause, for example, damage to cell membranes, disruption of the synthesis of some proteins or their degradation, or cause the formation of reactive oxygen species [18]. Gram-positive bacteria are more sensitive to silver than Gram-negative bacteria, which results from the fact that the presence of more layers of peptidoglycan reduces sensitivity of these cells. In addition, studies have shown that the formation of AgNPs combinations with commonly used antibiotics increases their effect [3,19,20]. Due to their antimicrobial properties, AgNPs are popular agents used in production of cleaning and disinfecting agents for surfaces and textiles, gels, or antimicrobial paints, as well as food packaging and preservation materials. They can be found in many daily used products, such as clothing, toothpastes, deodorants, and other antibacterial cosmetics, and even in the interior of refrigerators and washing machines [10,21]. However, toxic effects of AgNPs on various organisms, including humans, and on the environment have been reported recently. The mechanism of silver toxicity is not well understood yet, and requires further research in this area [10,22,23,24,25,26,27].

Among the methods allowing the synthesis of AgNPs, the three main groups are distinguished [28]. The most common are chemical methods, due to their relative ease and efficiency, where various organic solvents are used [17]. Another group are physicochemical methods, in which specialized equipment is often used, because the synthesis is carried out using ultrasounds or irradiation. The last group includes biological methods that are based on the use of plant extracts, vitamins, and amino acids, or the use of bacteria and fungi [5,18,29,30].

Here we report synthesis of AgNPs which by design are characterized as biologically inactive. That was achieved by applying the low-temperature synthesis in the presence of tannic acid followed by exhaustive purification by multiple centrifuging and ultrasound-assisted dissolving. The hydrogels were prepared from C_12_Ala with an addition of sodium citrate stabilizing additive, while AgNPs-doped hydrogel was prepared from AgNPs stock solution, solid C_12_Ala, and sodium citrate solution.

Biological activity of sole silver nanoparticles as well as in the combination with gelator were assessed against a panel of microorganisms including bacteria and fungi tumor and nontumor human cell lines, human red blood cells, and zebra-fish embryos.

## 2. Results and Discussion

### 2.1. AgNPs Synthesis

AgNPs stabilized by citric acid, with controlled dimensions and surface properties, were obtained using a modified method published in our previous work [31]. The quoted procedure consists of a reduction of ionic silver to metallic nanoparticles in an aqueous system with citric acid. In this reaction, the reducing agent was also used as a capping ligand generating a stabilizing, negative charge on the surface of the obtained AgNPs. The main factors affecting the kinetics of reduction, nucleation, and then the aggregation of growing seeds are concentration of reagents, reaction time, and also the temperature [32]. Each of these has a strong impact on morphological and physicochemical, as well as antibacterial, properties of the obtained nanomaterials. Typically, AgNPs are prepared in boiling aqueous solution by reduction of soluble silver salts by NaBH_4_ or other mild reductant. The low-temperature syntheses are rare but they certainly may lead to AgNPs of a different structure and surface properties. This option was selected and the AgNPs were obtained at a room temperature in sodium citrate and tannic acid, physically purified, and dissolved in water to obtain a solution of 4.4 mg/mL concentration.

**Figure 1:**
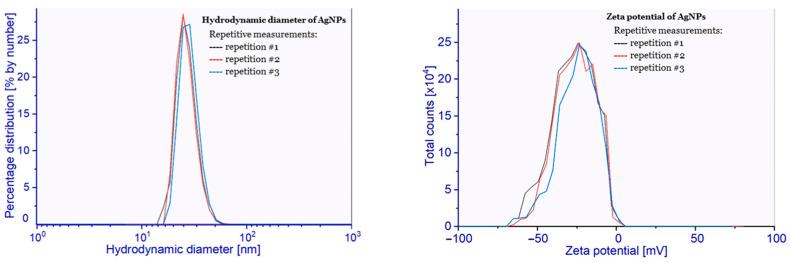
(**Left**) Hydrodynamic diameter distribution and (**Right**) surface zeta potential of AgNPs (the different colors correspond to repeated measurements).

Physicochemical characteristics of AgNPs were determined using the DLS method, SEM, and ultraviolet–visible absorption spectroscopy (UV–Vis). Furthermore, the composition of the AgNPs surface was analyzed by means of X-ray photoelectron spectroscopy (XPS). The hydrodynamic diameter of obtained AgNPs, determined by DLS, was equal to 24 ± 5 nm with PDI (polydispersity index) of 0.04. The particle size and the PDI indicated the absence of aggregates in the tested suspension. The zeta potential of obtained nanoparticles, calculated to be −26 ± 6 mV, reveals high stability of the tested AgNPs suspension (at pH = 6.67), with a high negative surface charge resulting from the presence of dissociated citric acid molecules adsorbed on the surface of nanoparticles. The presence of a high surface charge effectively inhibits the process of aggregation of nanoparticles, which is observed in DLS (hydrodynamic diameter) analysis. The measurement results are shown in Figure 1.

**Figure 2:**
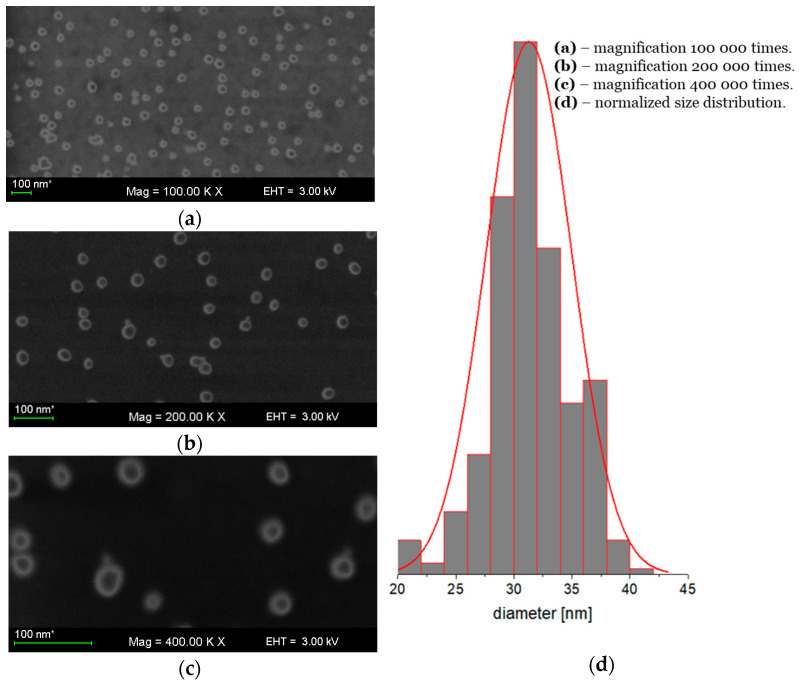
SEM images of AgNPs nanoparticles monolayer on PEI-modified silica wafers with various magnifications, and their normalized size distribution at N = 500 (D). (**a**) 100 KX; (**b**) 200 KX; (**c**) 400 KX; (**d**) AgNPs size distribution.

The AgNPs monolayer for SEM observations was obtained by the diffusion–adsorption method [33] according to the random sequential adsorption (RSA) theory [34,35]. The AgNPs monolayer adsorbed on the polyethyleneimine (PEI)-modified silicon surface is shown in various magnifications in Figure 2a–c. The normalized Gauss distribution of diameters calculated by graphical analysis (ImageJ software) of SEM images is shown in Figure 2d. The average diameter of AgNPs determined by SEM is 31 nm. The difference in values of hydrodynamic diameter measured by the DLS and SEM methods is naturally explained by specificity of the techniques used [36].

**Figure 3:**
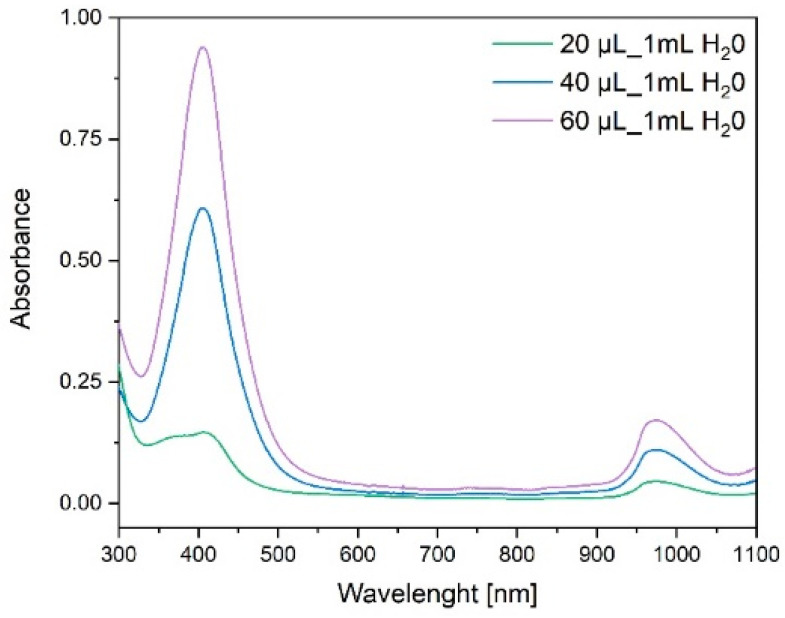
UV–Vis absorption spectroscopy of silver nanoparticles solutions.

The electronic absorption spectra of the obtained AgNPs, as presented in Figure 3, show a characteristic intense narrow band at λ_max_ = 400 nm and an additional band at λ_max_ = 1000 nm. The stock solution of AgNPs with concentration of 1.9 mg/mL was used for preparation of more diluted solutions (20, 40, and 60 µL of stock solution per 1 mL of water) for UV–Vis spectroscopy.

**Figure 4:**
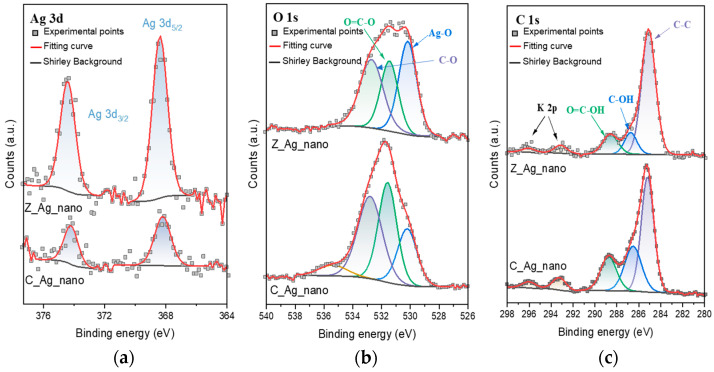
XPS core level spectra of (**a**) Ag 3d, (**b**) O 1s, and (**c**) C 1s for the AgNPs.

AgNPs were isolated from the suspension and, after drying, examined for the surface composition using the XPS method (Figure 4). Core level spectra of Ag 3d, O 1s, and C 1s are shown in Figure 4. The collected results confirm that the stabilizer remains on the surface of the nanoparticles. This is evidenced by the peaks at E_b_ = 285.1 ± 0.1 eV (C–C species), 286.6 ± 0.2 eV (C–OH species), and 288.7 ± 0.1 eV (carboxyl species) observed in the XPS C 1s spectra, as well as at E_b_ = 531.5 ± 0.1 eV (O=C–O species) and 532.7 ± 0.1 eV (C–O species) in the XPS O 1s spectra [37]. In the discussed areas, we also found the features that confirm the presence of water adsorbed on the surface (E_b_ = 535.4 eV), as well as trace amounts of K (doublet with K 2p_3/2_ at 293.1 eV and K 2p_1/2_ at 296.0 eV). The surface also contains silver, but its chemical state may not be uniform. Both samples show the Ag 3d_5/2_ peak at 368.3 ± 0.1 eV and the Ag 3d_3/2_ peak at 374.3 ± 0.1 eV. Such high binding energy value of these peaks may indicate the presence of metallic silver (oxide systems usually give peaks at E_b_ below 368 eV) [38,39]. On the other hand, in the O 1s region, the component typical for the emission of photoelectrons from oxygen atoms bound to metal atoms (Eb = 530.2 eV) is observed. Most likely, therefore, the surface of silver is partially oxidized to form film of silver hydroxide or silver doped with oxygen atoms [40,41,42]. Notably, the presence of oxygen atoms bonded to silver was not evidenced in the case of highly biologically active silver nanoparticles prepared in regular hydrothermal conditions [31].

### 2.2. Preparation of Hydrogels

Inspired by our previous results in the preparation of a stable and nontoxic gold nanoparticle containing hydrogels [11] suitable for medical applications and drug delivery, we decided to use the same gelator C_12_Ala which had been thoroughly studied and proved to be efficient and safe [11,43,44]. The blank hydrogel containing C_12_Ala was prepared as a reference. At first, we measured the solubility of C_12_Ala in water at 20 °C. The experiments were based on a direct measurement of a weight loss of insoluble material recovered after ultrasonic-assisted dissolving of excess C_12_Ala in water at 30 °C, and 4 days equilibration at 20 °C without the ultrasonic irradiation. The measured solubility was determined as 116 mg/L. The development of stable hydrogels required formation of the net of fibers of gelator from the permeated solution. We found that hydrogels containing less than 5% of the gelator undergo relatively fast phase transformation and precipitation of gelator. The stable gels were obtained after rapid cooling of (prepared at 85 °C in ultrasonic bath) 7% solution of C_12_Ala in 0.5% aqueous solution of sodium citrate. The C_12_Ala is the molecule with polar and nonpolar parts. In the presence of a solvent, the gelator molecules connect with each other with the polar parts through weak hydrogen bonding and electrostatic interactions [44], forming thin and long fibers of the gel matrix. In the case of strongly hydrophilic molecules dissolved by polar solvents, the structures of the gel matrix look very different. ZhangPing Li and coworkers showed SEM images of the internal structure of a hydrogel made of polyethyleneimine-modified pectin, as seen in Figure 2 in [45].

The hydrogels doped with AgNPs (C_12_Ala-AgNPs) were obtained similarly from the solution of AgNPs (4.4 mg/mL and 2.2 mg/mL), sodium citrate (0.00125 M), and solid C_12_Ala. Formed hydrogels were examined by applying several physicochemical methods.

### 2.3. SEM Analysis

**Figure 5:**
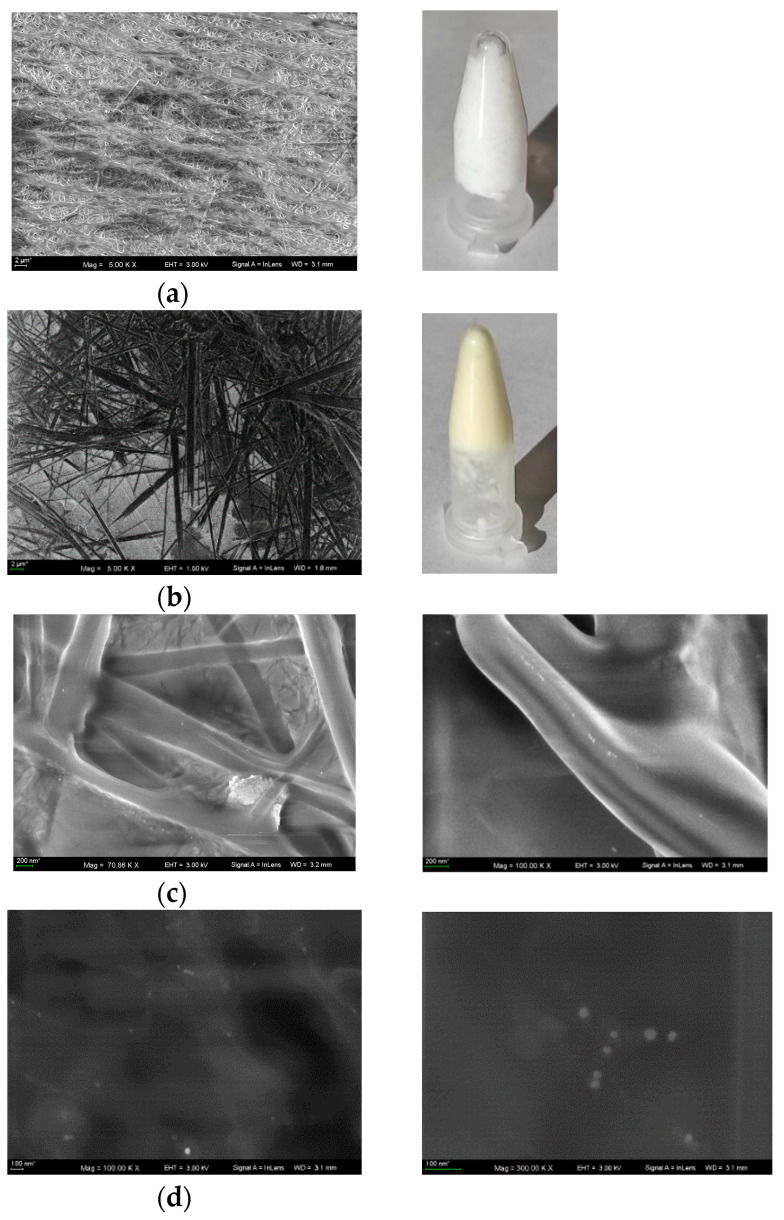
The SEM images of hydrogels. (**a**) hydrogel without AgNPs; (**b**) hydrogel with AgNPs; (**c**) SEM images of AgNPs distributed among the fibers of gel in two magnificstions; (**d**) SEM images AgNPs in melted gel in two magnificstions.

The SEM images of hydrogels stored for 5 months at 5 °C presented in Figure 5 correspond to the blank hydrogel (Figure 5a); C_12_Ala-AgNPs containing 4 mg/L of AgNPs (Figure 5b); AgNPs distributed among the fibers of gel, and the melted by electron beam hydrogel containing AgNPs (Figure 5c); and separated AgNPs in completely melted gel (Figure 5d). 

As it was measured, the average diameter of gel fibers in blank hydrogel was between d = 0.15–0.25 µm, while in hydrogel doped with silver, it was between d = 0.6–1.8 m. This could be rationalized by the assumption that the negatively charged AgNPs most strongly interact with thinner nanofibers and promote their recrystallization and fusion. The metal nanoparticles also make the hydrogel less stable under the electron beam irradiation, and the gelator fibers are melted during the measurement.

**Figure 6:**
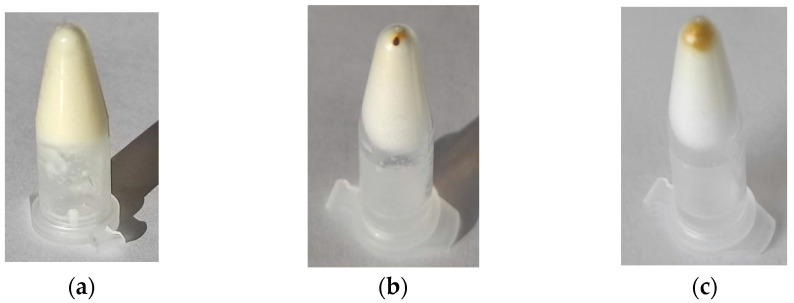
C_12_Ala-AgNPs (**a**) before, (**b**) after rotation, and (**c**) after 24 h of storing.

We confirmed a weak interaction of AgNPs with fibers of the gelator in centrifuge experiment. The AgNPs were separated from the gel; after 15 min of rotation at 13,000 rpm, the majority of AgNPs were collected at the bottom of the centrifuge vessel (Figure 6a), and they were dissolved back after storing for 24 h at ambient temperature (Figure 6c).

### 2.4. Thermal Resistance

As was already demonstrated, the presence of AgNPs in the liquid phase located between the nanofibers affected the properties of gel and its stability. More information about this phenomenon was gathered during thermal analysis.

#### 2.4.1. The Melting and Crystallization Point of Gels

**Figure 7:**
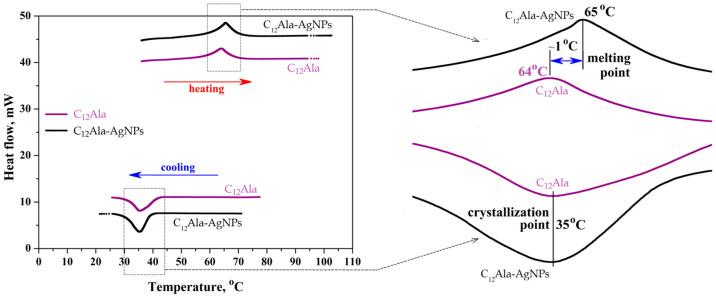
The second cycle (heating and cooling) of DSC measurements of C_12_Ala and C_12_Ala-AgNPs gel samples.

The three repetitions of the gel samples’ heating and cooling process were performed in the temperature range from 20 °C to 100 °C by DSC method. Figure 7 shows the second repetition for both samples.

The melting point is clearly visible in Figure 7 at around 65 °C and it fluctuates by one degree between the pure sample C_12_Ala gel and the sample doped with silver nanoparticles, C_12_Ala-AgNPs. The crystallization point of the sample does not depend on the nanoparticle content and amounts to 35 °C. The results of the DSC test indicate that the gel samples are stable in the tested temperature range; they do not change their chemical characteristics in the multicortical dissolution and gelling process. Moreover, the presence of silver nanoparticles does not significantly affect the stability of the gel matrix.

#### 2.4.2. Temperature Decomposition of Gels

**Figure 8:**
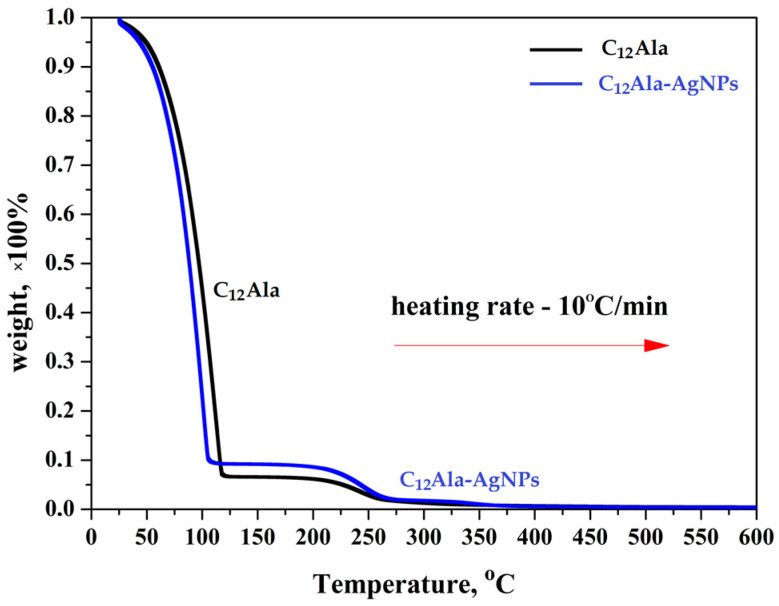
TGA curves of C_12_Ala and C_12_Ala-AgNPs gel samples.

The total decomposition of the gels was recorded at a temperature of about 350 °C. In comparison, the total decomposition temperature of analogical gel enriched with gold nanoparticles (C_12_Ala-AuNPs) was about 300 °C (see Figure 2 in [11]). Figure 8 shows thermograms for C_12_Ala gel and C_12_Ala-AgNPs gel samples, obtained for a heating rate of 10 °C/min.

**Figure 9:**
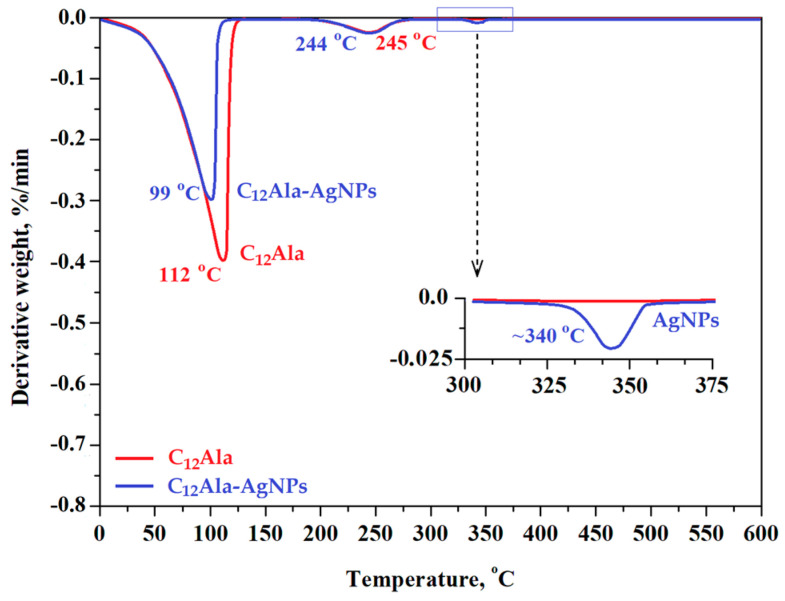
Temperature dependence of the derivative of weight loss on the heating rates of C_12_Ala and C_12_Ala-AgNPs gels.

As can be seen in Figure 8, the very fast weight loss (from 100% to ~10% of weight) was recorded for both samples in the temperature range from 20 °C to 125 °C. This process is related to the rapid evaporation of the solvent from the gels. In the temperature range from 125 °C to 275 °C for both samples, another process of a mass loss occurs, related to the decomposition of the gelator C_12_Ala. Moreover, as shown in the graph of the TGA derivative (Figure 9), over the temperature range of 330–350 °C, an additional peak is clearly visible. This peak is related to the degradation of the silver particles (AgNPs) and suggests that the silver nanoparticles are not chemically bound to the C_12_Ala gelator molecules. In the case of strong bonding of nanoparticles to gelator fibers, the decomposition peak for nanoparticles would not be so pronounced; nanoparticles would decompose together with gelator fibers. The TGA measurements were performed with heating rates of 1 °C/min, 5 °C/min, and 10 °C/min to calculate the kinetics of samples decomposition by the Ozawa–Flynn–Wall (OFW) theory [46]. The results (not shown in this work) were similar to the sample enriched with gold nanoparticles AuNPs. Within the limit of 10% liquid phase loss, we found that C_12_Ala-AgNPs can stay at a temperature of 10 °C for about 65 min, whereas increasing the temperature to 90 °C shortens this time to only a few minutes. For C_12_Ala-AuNPs, the limit of 10% of weight at 10 °C was reached after 70 min [11].

In general, the thermal analysis proves the stability of hydrogels at ambient temperature and the lack of strong interaction between AgNPs and C_12_Ala gelator.

### 2.5. Biological Activity

#### 2.5.1. Antimicrobial Properties

In the context of the biological activity of silver, the antimicrobial properties of the metal are the most common. Newly synthesized at low temperature AgNPs were examined by the broth microdilution method against twelve bacteria and one fungal strains. Surprisingly, the AgNPs at the tested concentration of up to 1 mg/mL did not inhibit the growth of any of the tested microorganisms, neither bacteria nor fungi (Table 1). The results are in opposition to these presented by others, where AgNPs were obtained in the hydrothermal synthesis. Hwang et al. also tested antibacterial activity of AgNPs against human pathogenic bacteria, including two strains of *E. coli* and *P. aeruginosa, S. aureus*. Nanoparticles showed antibacterial activity with MIC (minimal inhibitory concentration) values of 0.5–2 µg/mL [20]. In research by Pucelik et al. on multifunctional silver nanoparticles modified with 11-mercapto-*N*, *N*, *N*-trimethylammonium chloride (TMA), and sodium citrate (TSC), MIC values of 0.17 μg/mL and 0.05 μg/mL, respectively, for *E. coli* and *S. aureus* were obtained [31]. AgNPs were also the subject of the research of Loo et al. [47]. In their study, the MICs of AgNPs against *E. coli*, *K. pneumoniae*, *S. typhimurium*, and *S. enteritidis* were 7.8, 3.9, 3.9, and 3.9 μg/mL, respectively. In antimicrobial tests performed by Liao et al., AgNPs were shown to have a strong bactericidal effect on drug-resistant and multidrug-resistant *P. aeruginosa* with an MIC range of 1.4–5.6 µg/mL [48]. Antimicrobial studies on the gelator C_12_Ala and its variant containing AgNPs showed a lack of both antibacterial and antifungal properties (Table 1).

**Figure 10:**
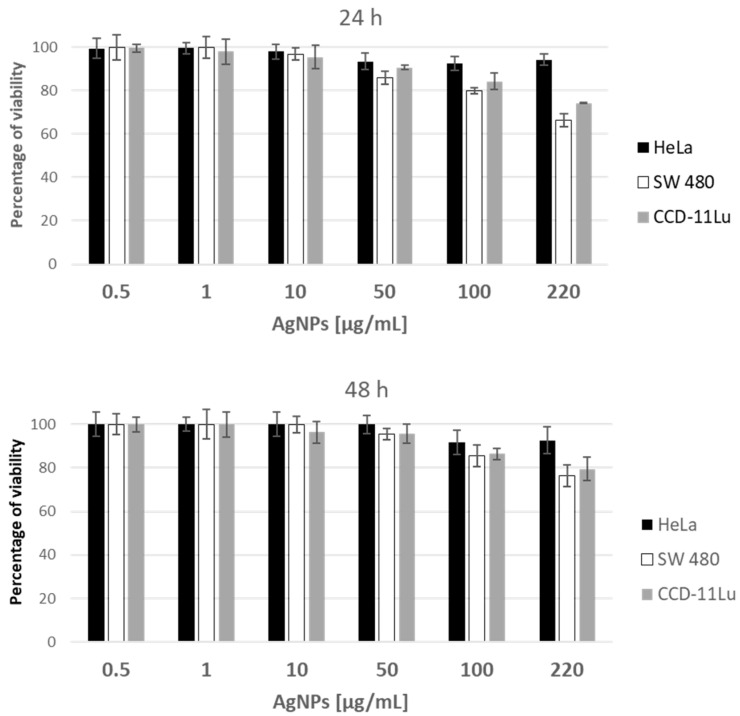
Viability (%) of human cell lines exposed to the increasing concentrations of AgNPs, assessed with the use of (3-[4,5-dimethylthiazol-2-yl])-2,5 diphenyl tetrazolium bromide (MTT) assay after 24 and 48 h of incubation.

In addition to antimicrobial properties, silver nanoparticles are known for their anticancer activity. The synthesized AgNPs have been tested for anticancer activity against normal (CCD-11Lu) and cancer (HeLa, SW480) human cell lines. At the lowest tested concentration of 0.5 µg/mL against HeLa, SW480, and CCD cells, cell viability was very close, or equal, to 100%. However, at the highest concentration (220 µg/mL) for HeLa cell line, AgNPs inhibit the proliferation only by 5.73% after 24 h of incubation and by 7.41% after 48 h. In the case of the SW480 and CCD-11Lu lines, the inhibition of proliferation was higher and amounted to 23.54% and 20.5% after 48 h of incubation, respectively (Figure 10). 

Similarly to antimicrobial inactivity, the AgNPs show the minor influence on human cancer cells only at the highest tested concentration, which does not correspond with the results obtained by others, where hydrothermal AgNPs synthesis was applied. Pucelik et al. found that AgNPs are highly toxic to cancer cells [31]. The studies included AgNPs of different sizes (DLS: 3.1–41 nm) with positive surface charge: Ag@TMA1, Ag@TMA2, and negative surface charge: Ag@TSC1, Ag@TSC2 against human keratinocytes (HaCaT) and cancer cell line: CT26, 4T1. The test included the concentration of 0.1 and 0.5 µg/mL and incubations for 2 and 24 h. In concentration of 0.50 μg/mL, AgNPs show high toxicity in all tested cases. Other studies on the cytotoxicity of AgNPs were carried out against breast cancer cell lines (MCF-7) and human lung fibroblast cell lines (WI38). The IC_50_ of cellular inhibition of silver nanoparticles was observed at 27.79 ± 2.3 and 31.78 ± 2.2 µg/mL, respectively [49]. On the other hand, studies on biosynthesized silver nanoparticles showed reduced cell viability in colon cancer cells (HCT-116) at an IC_50_ concentration of 100 μg/mL [50]. Next, the gelator alone, as well as in the combination with AgNPs, was tested against two cancer and one nontumor human cell lines. The presented high IC_50_ values, starting from 1.86 mg/mL revealed low anticancer activity of these substances (Table 2). In spite of such results, firstly, nontumor CCD-11Lu cells showed lower susceptibility to gelators, and, secondly, addition of AgNPs resulted in higher cytotoxicity, especially against tumor SW480 cell line.

#### 2.5.2. Hemolytic Properties

**Figure 11:**
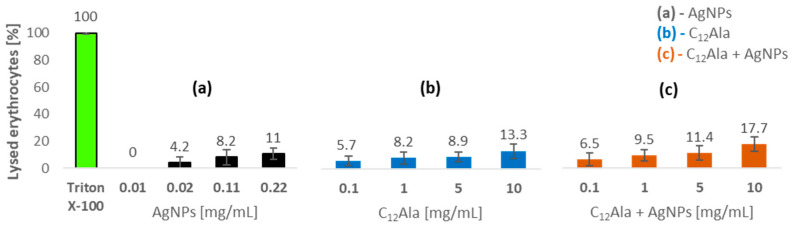
Hemolytic activity of AgNPs and gelators measured by percent of lysed erythrocytes compared to control (Triton X-100). (**a**) AgNPs; (**b**) C_12_Ala; (**c**) C_12_Ala-AgNPs.

In order to confirm the low toxicity of metal nanoparticles against normal human cells, AgNPs alone, as well as in the combination with the gelator, were tested for hemolytic activity using human erythrocytes (Figure 11). At the concentration of 11 µg/mL, the nanoparticles do not cause hemolysis of the blood cells. In turn, at the concentrations of 22 and 110 µg/mL, 4.2% and 8.2% of the blood cells were lysed, respectively. The highest percentage of hemolysis (11%) was obtained at the highest concentration (220 µg/mL). The results show that the AgNPs, even at high concentration, have little hemolytic activity. Korolev et al. [51] and Zharkova and team [52] obtained similar results. They found that the hemolytic activity of unmodified AgNPs was minimal and did not exceed a few percent. In turn, Chen et al. [53] investigated the hemolytic activity against three diameters of AgNPs. Studies showed that AgNPs15 resulted in a much higher level hemolysis (60%) compared to AgNPs50 and AgNPs100, which caused less than 12% hemolysis even at the highest tested concentration. The combination of AgNPs with the gelator results in slightly higher hemolysis in comparison with an activity of sole C_12_Ala, but taking into consideration that gels were tested at quite high concentrations, this effect was rather irrelevant (Figure 11).

#### 2.5.3. Ecotoxic Properties

**Figure 12:**
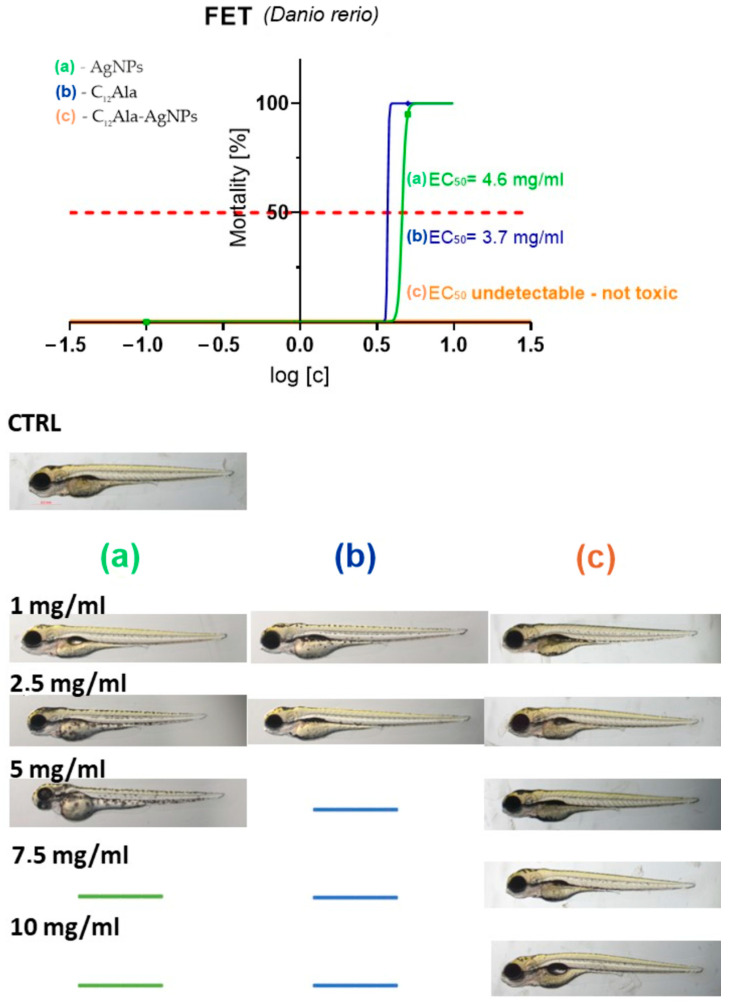
Determination of the fish embryo toxicity (FET), expressed as EC_50_ values, and the effect of the tested compounds on the morphology of zebra-fish embryos. (**a**) AgNPs, (**b**) C_12_Ala, (**c**) C_12_Ala-AgNPs.

In the final stage, silver nanoparticles and gelators were tested for ecotoxic properties using the in vivo *Danio rerio* model (Figure 12). 

C_12_Ala-AgNPs was not toxic at all, while compounds AgNPs and C_12_Ala were toxic at the dose of 5 mg/mL. No malformations were observed. The toxicity of AgNPs and C_12_Ala were exerted in coagulation of fertilized eggs in concentrations of 5 mg/mL and higher.

## 3. Materials and Methods

### 3.1. General CPL

AmpB, TSC, silver nitrate, tannic acid, and PEI (branched, analytical standard) were purchased from Sigma-Aldrich Chemicals, St. Louis, MO, USA. Distilled water was obtained with Millipore system (ρ = 18.2 MΩ) and used in all experiments and for washing glass parts of the laboratory equipment. Silicon wafers used in the experiments were purchased from Siegert Wafer GmbH (Aachen, Germany). XPS measurements were performed in a Prevac photoelectron spectrometer consisting of an analysis UHV chamber with a hemispherical analyzer (VG SCIENTA R3000 (Pleasanton, CA, USA)). A monochromatized aluminum source Al Kα (hν = 1486.6 eV) was used for acquiring survey- and cor-level spectra. The scale of binding energy value was calibrated by referring to a position of Au 4f (E_b_ = 84.0 eV). Curve-fitting analysis of all spectra was performed in the Casa XPS software using the mixed Gauss and Lorentz function (GL = 30) after subtraction of the Shirley-type background. The reference strains of microorganisms from Polish Collection of Microorganisms (PCM) of Hirszfeld Institute of Immunology and Experimental Therapy of Polish Academy of Sciences (Wrocław, Poland), and from American Type Culture Collection (ATCC) (Manassas, VA, USA) were included. The representative Gram-positive bacteria were *Staphylococcus aureus* ATCC 6538, *Staphylococcus aureus* ATCC 25923, *Staphylococcus aureus* ATCC BAA-2313 (MRSA), *Staphylococcus epidermidis* PCM 2651, and *Streptococcus pneumoniae* PCM 2589, while the Gram-negative bacteria were *Acinetobacter lwoffii* PCM 2235, *Escherichia coli* ATCC 25922, *Proteus vulgaris* PCM 1347, *Klebsiella pneumoniae* PCM 1, *Pseudomonas aeruginosa* PCM 3035, *Enterobacter cloacae* PCM 2569, and *Enterococcus faecalis* PCM 2786. Moreover, the fungi belonging to yeasts, *Candida albicans* ATCC 10231, were used. The CCD-11Lu (normal lung fibroblasts), HeLa (cervix adenocarcinoma), and SW480 cell line (colorectal adenocarcinoma) were obtained from ATCC. DSC measurements were made using a DSC 4000 (−50–400 °C)/PERKIN ELMER differential calorimeter. TGA measurements were made on a TGA 8000 (−20–1200 °C)/PERKIN ELMER. The hydrodynamic size distribution and surface zeta potential of the manufactured solutions were assessed directly through the DLS technique via the diffusion coefficient measurements conducted using ZetaSizer Nano ZS Instrument (Malvern Panalytical, Malvern, UK) from Malvern. The measurements were conducted using appropriate quartz cuvettes, dedicated to such measurements. The analysis of the statistical distribution of the nanoparticles’ sizes was carried out through computing SEM images. To evaluate the NPs size distribution, an analysis software for graphical images was implemented: ImageJ (https://imagej.nih.gov/, accessed on 26 December 2022). The statistical analysis was conducted for a probe of N = 500. UV–Vis spectroscopy was implemented to examine the solutions of manufactured nanoparticles in the wavelength range of 300–1100 nm, using a UV-2600 spectrometer (Shimadzu, Kyoto, Japan). The measurements were conducted using appropriate quartz cuvettes, dedicated to such measurements. The weight concentration of silver (Ag) nanoparticles was measured by a densitometer DMA 5000M from Anton Paar. The SEM measurements were recorded on a ZEISS Ultra Plus microscope.

### 3.2. AgNPs Preparation

Silver nanoparticles with controlled dimensions and surface properties were obtained by a modified method published in our previous work [31]. A total of 20 mL of 25 mM solution of TSC was mixed with 2 mL 5 mM solution of tannic acid and diluted with 78 mL of water. The obtained solution was stirred for 15 min at room temperature (RT), then 3 mL of 25 mM solution of AgNO_3_ was added dropwise. The change in the color of the solution, from bright yellow through intensive yellow to slightly red, within several minutes indicated the formation of AgNPs coated with the citric ligands. After the reaction, the final solution was purified by recentrifugation at 2500 rcf, which led to the separation of NPs larger than 40 nm from the supernatant. The purification process was carried out by recentrifugation at 18,000 rcf and then the solution was washed several times, resulting in a precipitate of AgNPs with water. The size and zeta potential of the final AgNPs were estimated by DLS and SEM. The concentration of the obtained suspension was determined by density measurement, which was 4.4 mg/L.

#### Determination of Concentration of AgNPs

The weight concentration of silver (Ag) nanoparticles in the solution after purification was evaluated by measuring the density of the native suspension *ρ*_s_ and the effluent *ρ*_e_ obtained following centrifugation. The mass concentration is then determined using the formula [54]:

$$w=\frac{\rho_{p}\left(\rho_{s}-\rho_{e}\right)}{\rho_{s}\left(\rho_{p}-\rho_{e}\right)}$$

where: $\rho_{p}=10.49\;g/{\mathrm{cm}}^{3}$ is the specific density of silver nanoparticles [55].

According to the discussed procedure, the concentration of AgNPs was found to be 4.4 mg/L.

### 3.3. Silicon Substrate Treatment for NPs Microscopy Observations

The procedure is based on thoroughly washing the silicon substrate (1 cm × 1 cm) in methanol and then in ultrapure water under sonication treatment. In the next step, ultraclean and dried under argon flush substrates were oxidized by piranha solution (3 parts of concentrated sulfuric acid and 1 part of 30% hydrogen peroxide solution; 15 min). The PEI polyelectrolyte monolayer was applied onto the surface of the silicon substrate by the layer-by-layer LBL technique. In the case of the negative charge of NPs, one PEI layer was formed on the surface, as a result of immersing substrates in a PEI solution (1 mg/mL; 30 min). The deposition of the NPs was carried out by immersing the substrates in the nanoparticles’ dispersion during 20 min. After this process, the substrates were thoroughly washed with water and dried with argon. Freshly prepared solutions of polyelectrolytes, which were previously sonicated, were used.

### 3.4. Preparation of the Hydrogels, Typical Procedure

The dry glass vial equipped with the stirrer magnet was charged with 7.3 mL of solution AgNPs (4.4 mg/mL), 560 mg of solid C_12_Ala was added, and after 2 min of stirring, 0.12 mL of a 1.25 M solution TSC was added. The vial was closed and the reaction mixture was heated at 85–90 °C in ultrasonic bath for 10 min to achieve the homogenous mixture. Next, the vial was cooled down to 0–5 °C in an ice bath without stirring. After the cooling was completed, the stable at ambient (and lower) temperature light yellow gel was formed, as presented in Figure 5B.

### 3.5. Differential Scanning Calorimetry (DSC)

DSC measurement was used to analyze phase transitions related to temperature change in samples and determine the phase and structural changes taking place in the sample during heating along with their kinetics, exothermic and endothermic chemical reactions, changes in the heat capacity of the sample, and, in some cases, as well as the temperature stability of the samples.

### 3.6. Thermal Gravimetric Analysis (TGA)

TGA was used to record changes in the mass of a sample during its thermal decomposition to make conclusions regarding the amount of thermal transformation and the temperature at which this transformation takes place.

### 3.7. Determination of Minimum Inhibitory Concentrations (MICs)

The substances were investigated in vitro for antimicrobial activities. In these studies, the broth microdilution was used. The MIC of the substances was evaluated by the microdilution broth method in 96-well polystyrene plates. The tests were performed in accordance with the guidelines of the European Committee on Antimicrobial Susceptibility Testing (EUCAST) [56] and Clinical and Laboratory Standards Institute (CLSI) [57]. All the used microbial cultures were first subcultured on nutrient agar (for bacteria) or Sabouraud agar (for *Candida*) (BioMaxima S.A., Lublin, Poland). Twofold dilutions of the tested compounds in selective Mueller–Hinton broth (BioMaxima S.A., Lublin, Poland) for bacteria and RPMI 1640 (Sigma-Aldrich Chemicals, St. Louis, MO, USA) with MOPS (3-(N-Morpholino)propanesulfonic acid) (Sigma-Aldrich Chemicals, St. Louis, MO, USA) for fungi were performed. The final concentrations of AgNPs ranged from 1000 µg/mL to 0.35 µg/mL, C_12_Ala ranged from 6000 µg/mL to 11.7 µg/mL, and C_12_Ala + AgNPs ranged from 6000 µg/mL to 11.7 µg/mL. Bacterial and fungal suspensions were prepared in sterile NaCl with an optical density of 0.5 McFarland standard. Next, the microbial suspension was introduced into each well of the microplate containing broth and various concentrations of the tested substances. After 24 h incubation, the MIC value was assessed visually as the minimal concentration of the samples that showed complete microbial growth inhibition. Each concentration range for all microbial species was tested in triplicate. Appropriate DMSO, sterile, and growth controls were performed. The media with and without tested substances/DMSO were used as controls. Standard antibiotics, antibacterial CPL, and antifungal AmpB were used as reference substances.

### 3.8. Cell Culture

The CCD-11Lu (normal lung fibroblasts), HeLa (cervix adenocarcinoma), and SW480 cell line (colorectal adenocarcinoma) cells were cultured in DMEM (Dulbecco’s Modified Eagle Medium, high glucose)+ GlutaMAX supplemented with penicillin (100 U/mL), streptomycin (100 U/mL), and 10% heat-inactivated fetal bovine serum (FBS). Cells were maintained in a humidified atmosphere at 37 °C and 5% CO_2_ and passaged twice before performing an experiment.

### 3.9. Antiproliferative Assay

For antiproliferative assay, cells of each line were seeded in 96-well microplates (Wuxi Nest Biotechnology, Wuxi, Jiangsu, China) at a density of 2.5 × 10^4^ cells/mL in 100 μL DMEM + GlutaMAX supplemented with 10% heat-inactivated FBS in two sets for different periods of tested compound exposure. After 24 h of cell attachment, plates were washed with 100 μL/well of Dulbecco’s phosphate-buffered saline (DPBS) and the cells were treated with increasing concentrations (0.0005–0.22 mg/mL final concentration) of AgNPs and 0.056–100 mg/mL final concentration of C_12_Ala and C_12_Ala-AgNPs prepared in fresh FBS-free medium for 24 and 48 h. All stock solutions were diluted to the desired concentration with the culture medium only. Each concentration was tested in triplicate. Cytotoxicity of compounds was assessed using (3-[4,5-dimethylthiazol-2-yl])-2,5 diphenyl tetrazolium bromide (MTT) assay as described below. Following 24 and 48 h of compound exposure, control medium or test exposures medium were removed, the cells were rinsed with DPBS, 100 μL of fresh medium (without FBS or antibiotics) containing 0.5 mg/mL of MTT was added to each well, and the plates were incubated for 3 h at 37 °C in a 5% CO_2_ humidified incubator. After the incubation period, the medium was discarded, the cells were washed with 100 μL of DPBS, and 100 μL of DMSO was added to each well to extract the dye. The plate was shaken for 10 min and the absorbance was measured at 570 nm. Viability was calculated as the ratio of the mean of OD obtained for each condition to the control condition. Calculations of IC_50_ values and statistical analysis were performed in GraphPad Prism 9 software.

### 3.10. Hemolytic Activity

The hemolytic activity of the AgNPs and gelators was determined on human red blood cells. Human erythrocytes were harvested by centrifugation for 10 min at 2000 rpm and 4 °C. Then, the pellets were washed in PBS buffer (10 mM phosphate, pH 7.5; 150 mM NaCl). This step was performed three times. The resulting suspension was serially diluted 10-fold in PBS. A further 1:10 dilution of PBS was prepared from this suspension. Then, 450 μL of the final diluted erythrocytes were added to microcentrifuge tubes, which contained test compounds in an amount of 50 µL each. The final concentrations was 11–220 µg/mL (AgNPs), and 0.1–10 mg/mL (C_12_Ala and C_12_Ala + AgNPs). Test concentrations were prepared by dissolving in PBS, and 1% Triton was used as a positive control, causing total hemolysis. The tubes were incubated at 37 °C for 1 h and then centrifuged for 10 min at 2000 rpm and 4 °C. A total of 150 µL of each tube was transferred to a flat-bottomed microtiter plate. The absorbance was measured at 450 nm. The hemolysis percentage was calculated by the following equation: % hemolysis = (A_450_ of test compound treated sample − A_450_ of buffer treated sample/ A_450_ of 1% Triton X-100 treated sample − A_450_ of buffer treated sample) × 100%. The experiments were performed in duplicate.

### 3.11. Ecotoxicity

To determine the toxicity of AgNPs, C_12_Ala, and C_12_Ala-AgNPs, the FET test was performed on zebra-fish (*Danio rerio*) according to OECD Test Guideline 236. Briefly, newly fertilized zebra-fish eggs were exposed to solutions of compounds for a period of 96 h at concentrations ranging from 1 to 10 mg/mL. The system applied in the experiment was static, as the changes of solution concentrations did not exceed the range of 20% of nominal concentration values.

The E3 solution (5 mmol/L NaCl, 0.17 mmol/L KCl, 0.33 mmol/L CaCl_2_, and 0.33 mmol/L MgSO_4_, containing no methylene blue, with a pH value of about 7.2) was used as embryo culture medium as well as to prepare the solutions. The experiment was performed in 24-well plates, with five embryos per well, ten per group, in three repetitions. Each plate was covered and kept in an incubator set at 28 ± 0.5 °C under a light/dark period of 12/12 h. At the end of the exposure period (96 hours post fertilization (hpf)), acute toxicity was determined based on a positive outcome in any of the four visual indicators of lethality, including the coagulation of fertilized eggs, lack of somite formation, lack of detachment of the tailbud from the yolk sac, and lack of heartbeat. The value of EC_50_ was calculated.

## 4. Conclusions

The main goal of this work was to prepare and investigate the properties of a new C_12_Ala-based hydrogel enriched with AgNPs in terms of its suitability for pharmaceutical purposes. On the basis of physical and biological tests, it was found that silver nanoparticles do not disturb the stability of the gel matrix, and both matrixes, pure and silver-enriched are stable in the temperature range of 20 °C to 100 °C. No interactions were found between gelator molecules and silver nanoparticles, which diffuse freely in the pores filled with the liquid phase of hydrogel.

In comparison with the biological activity of AgNPs published elsewhere, our nanoparticles show high level of biological neutrality when tested against microorganisms, human cells as well as zebra-fish embryos. Combination of AgNPs with the gelator C_12_Ala studied in parallel also showed trace biological activity towards the used models. Such biologically neutral assembly may be taken into consideration as promising agent for applications where side effects are unwanted, e.g., in drug delivery.

## Figures and Tables

**Table 1:** Assessment of antibacterial and antifungal properties of AgNPs, and C_12_Ala, as well as C_12_Ala-AgNPs, gels. MIC values expressed in µg/mL are presented. Chloramphenicol (CPL) and Amphotericin B (AmpB) were used as control antibacterial and antifungal agents, respectively.

Microorganism	AgNPs	C_12_Ala	C_12_Ala-AgNPs	CPL	AmpB
*S. aureus* ATCC 6538	>1000	>6000	>6000	15.6	n/a
*S. aureus* ATCC 25923				31.2	n/a
*S. aureus* ATCC BAA-2313				15.6	n/a
*S. epidermidis* PCM 2651				31.2	n/a
*S. pneumoniae* PCM 2589				15.6	n/a
*A. lwoffii* PCM 2235				7.8	n/a
*E. cloacae* PCM 2569				15.6	n/a
*E. faecalis* PCM 2786				15.6	n/a
*P. aeruginosa* PCM 3035				15.6	n/a
*E coli* ATCC 25922				15.6	n/a
*P. vulgaris* PCM 1347				15.6	n/a
*K. pneumoniae* PCM 1				15.6	n/a
*C. albicans* ATCC 10231				n/a	3.12

**Table 2:** IC_50_ (mg/mL) against tumor and nontumor cells at 24 h of incubation.

	HeLa	SW480	CCD-11Lu
**AgNPs**	>0.22	>0.22	>0.22
**C_12_Ala**	2.50 ± 0.25	3.08 ± 0.13	4.85 ± 1.28
**C_12_Ala + AgNPs**	2.33 ± 0.71	1.86 ± 0.32	4.35 ± 0.97

## Data Availability

The data supporting reported results can be received from the Authors.
